# CsBAFF, a Teleost B Cell Activating Factor, Promotes Pathogen-Induced Innate Immunity and Vaccine-Induced Adaptive Immunity

**DOI:** 10.1371/journal.pone.0136015

**Published:** 2015-08-21

**Authors:** Yun Sun, Li Sun

**Affiliations:** 1 Key Laboratory of Experimental Marine Biology, Institute of Oceanology, Chinese Academy of Sciences, Qingdao, 266071, China; 2 State Key Laboratory Breeding Base for Sustainable Exploitation of Tropical Biotic Resources, College of Marine Science, Hainan University, Haikou, 570228, China; 3 Function Laboratory for Marine Biology and Biotechnology, Qingdao National Oceanography Laboratory, Qingdao, China; COCHIN INSTITUTE, Institut National de la Santé et de la Recherche Médicale, FRANCE

## Abstract

B cell activating factor (BAFF) is a member of the tumor necrosis factor family that is known to play an important role in B cell activation, proliferation, and differentiation in mammals. However, studies of BAFF in teleosts are very limited and its function, in particular that under *in vivo* conditions, is essentially unknown. In this study, we conducted *in vivo* as well as *in vitro* functional analyses of a BAFF homologue (CsBAFF) from the teleost fish tongue sole (*Cynoglossus semilaevis*). CsBAFF is composed of 261 residues and shares moderate sequence identities with known BAFFs of other teleosts. *CsBAFF* expression was most abundant in immune organs and was upregulated during bacterial infection. Purified recombinant CsBAFF (rCsBAFF) bound to tongue sole lymphocytes and promoted cellular proliferation and survival. The results of an *in vivo* study showed that CsBAFF overexpression in tongue sole significantly enhanced macrophage activation and reduced bacterial infection in fish tissues, whereas knockdown of *CsBAFF* expression resulted in increased bacterial dissemination and colonization in fish tissues. Furthermore, vaccination studies showed that CsBAFF enhanced the immunoprotection of a DNA vaccine and augmented the production of specific serum antibodies. Taken together, these results provide the first *in vivo* evidence to indicate that teleost BAFF is an immunostimulator that significantly contributes to the innate antibacterial immune response and vaccine-induced adaptive immune response.

## Introduction

B-cell activating factor (BAFF), also known as BLys, TALL-1, THANK, zTNF4, and TNFSF13b, is a member of the tumor necrosis factor (TNF) family, and is mainly produced by innate immune cells, such as neutrophils, monocytes, and dendritic cells (DCs), as well as activated T cells and malignant B cells [1–7]. BAFF exists either as a type II transmembrane protein on the cell surface or a soluble protein after cleavage at the cell surface by a furin-like protease [1,2]. BAFF exerts its function by interaction with its receptor. To date, three BAFF binding receptors have been identified, i.e., transmembrane activator and CAML interactor (TACI), B-cell maturation antigen (BCMA), and BAFF-R [8,9]. TACI and BCMA can also bind to a proliferation-inducing ligand (APRIL), another member of the TNF family that shares a high level of sequence similarity with BAFF [10,11], while BAFF-R is specific for BAFF. Recent studies suggest that BAFF-R may be the principal receptor responsible for B-cell development and survival [9].

Several lines of evidence have indicated that BAFF is involved in the regulation and promotion of both innate and adaptive immune responses. In mammals, BAFF plays a major role in B cell survival, proliferation, and differentiation, and can modulate T cell function [8,12–16]. Previous studies have demonstrated that BAFF is required for T cell-independent type II responses and T cell-dependent immunoglobulin (Ig) M responses [14], and that BAFF collaborates with cytokines to promote IgG and IgA class switching and plasma cell differentiation [12,13]. Moreover, BAFF can also modulate memory B cells and their differentiation to plasma cells [15,16]. In addition, BAFF is associated with autoimmune diseases, such as systemic lupus erythematosus, rheumatoid arthritis, and primary Sjögren’s syndrome [17–19].

BAFF-like sequences have been identified in a number of teleost species, most of which, however, have not yet been studied in depth. To date, BAFF bioactivity has been documented in zebrafish (*Danio rerio*), yellow grouper (*Epinephelus awoara*), and fugu (*Takifugu obscurus*) [20–22], of which, in yellow grouper and fugu, BAFF promotes the survival/proliferation of splenic lymphocytes, similar to that of mouse splenic B cells *in vitro*. As a result of the limited number of studies, BAFF function in teleosts, particularly *in vivo*, remains largely unknown.

Tongue sole (*Cynoglossus semilaevis*) is an economically important teleost species in China. Although the cDNA sequence of the BAFF homologue of *C*. *semilaevis* (CsBAFF) has been identified, no relevant study on the *in vivo* function of CsBAFF has been conducted. The aim of the current study was to examine the *in vivo* and *in vitro* immune effects of CsBAFF, the former by creating a condition of CsBAFF overexpression or knockdown in *C*. *semilaevis*. On the basis of these investigations, we further investigated the potential of CsBAFF to enhance the immune response using a DNA vaccine.

## Materials and Methods

### Ethics statement

All protocols for experiments involving live animals conducted in this study were approved by the Ethics Committee of the Institute of Oceanology, Chinese Academy of Sciences (Shandong, China).

### Fish

Clinically healthy fish (free of prior infection and no clinical signs of infection or disease) were obtained from a fish farm in Shandong Province and reared at 20°C in aerated seawater for one week before being used for experimentation. Before conducting any experiment, the fish were confirmed to be free of common pathogens using methods reported elsewhere [23]. For experiments involving tissue collection, the fish were euthanatized with an overdose of tricaine methanesulfonate (Sigma–Aldrich, St. Louis, MO, USA) and then killed by severing the spinal cord.

### Sequence analysis

The cDNA and amino acid sequences of CsBAFF (GenBank accession number XM_008328682) were analyzed using the Basic Local Alignment Search Tool (BLAST) at the National Center for Biotechnology Information website (http://blast.ncbi.nlm.nih.gov/Blast.cgi) and the Expert Protein Analysis System (www.expasy.org), as reported previously [24]. Sequence alignment was performed using the ClustalX algorithm (http://www.clustal.org/).

### Quantitative real time reverse transcription-PCR (qRT-PCR)

Tissues (i.e., intestine, brain, gill, muscle, heart, blood, liver, kidney, and spleen) were collected aseptically from five tongue sole (average body weight, 14.4 ± 0.2 g). RNA extraction, cDNA synthesis, and qRT-PCR were performed, as reported previously [24]. *CsBAFF* expression levels were analyzed using β-actin as an internal control [24]. The experiment was performed independently three times. For bacterial infection, *Edwardsiella tarda* TX1 was grown in Luria–Bertani broth (LB) at 28°C to an optical density at 600 nm (OD_600_) of 0.8, as reported previously [25]. The bacterial cells were pelleted by centrifugation at 10,000g for 1 min at RT, washed with phosphate-buffered saline (PBS), and resuspended in PBS to a concentration of 2 × 10^6^ colony-forming units (CFU)/ml. Two groups of tongue sole (as above) received intramuscular injections of 100 μl of *E*. *tarda* suspension or PBS, respectively. At 6, 12, 24, and 48 h post-infection (hpi), kidney and spleen tissues were collected from five fish from each group. qRT-PCR was performed as described above with the 60S ribosomal protein L18a (for spleen) or β-actin (for kidney) as an internal control [24]. The experiment was performed independently three times.

### Preparation of recombinant proteins

To construct the plasmid pEtCsBAFF, which expresses His-tagged recombinant CsBAFF (rCsBAFF), the coding sequence of the TNF domain of CsBAFF (residues 112–260) was amplified with the primer pair BAFF-F1/BAFF-R1 (Table 1), and the PCR product was ligated within the plasmid pET259 [26] at the EcoRV restriction site. To construct pGEXH, which expresses His-tagged glutathione S-transferase (GST), a His-tag linker (5′-GATCCCCCGGGCACCACCACCACCACCACTAAC-3′) was inserted into pGEX-4T-1 (GE Healthcare, Piscataway, NJ, USA) between the BamHI and XhoI restriction sites. To prepare rCsBAFF and recombinant GST (rGST), competent *Escherichia coli* BL21 (DE3) cells (Tiangen Biotech Co., Ltd., Beijing, China) were transformed with pCsBAFF and pGEXH. The transformants were grown in LB medium at 37°C to an OD_600_ of 0.7 and then isopropyl-β-D-thiogalactopyranoside (1 mM) was added to the culture. The cells were grown at 18°C for 12 h and then pelleted by centrifugation at 10,000g for 5 min at RT. Cellular proteins were purified using Ni-NTA agarose (Qiagen, Inc., Valencia, CA, USA), as recommended by the manufacturer. After dialyzing in PBS overnight, endotoxin was removed from the proteins using the Quantitative Chromogenic Tachypleus Amebocyte Lysate for Endotoxin Detection Kit (Chinese Horseshoe Crab Reagent Manufactory Co., Ltd., Xiamen, China) using methods reported elsewhere [27]. After treatment, the endotoxin content in the proteins was reduced to 7 endotoxin units/ml. The proteins were concentrated and separated by sodium dodecyl sulfate-polyacrylamide gel electrophoresis, as reported previously [27].

**Table 1:** Primers used in this study.

Primer	Sequence (5’-3’) ^a^
BAFF-F1	GATATCATGACTATTGTGTCTCAGT (EcoRV)
BAFF-R1	GATATCAACCAGTTTGAAAGCG (EcoRV)
BAFF-F2	GATATCGCCACCATGACTATTGTGTCTCAGT (EcoRV)
BAFF-F3	ACTATTGTGTCTCAGTCCTGCTTA
BAFF-RTF1	GAGCCTCAGTTTGTCGTCCT
BAFF-RTR1	GAAACGTTGGCTGTGGAACG
CN-F1	CTTGCGTTTCTGATAGGCACCTA
CN-R1	TGCGGGCCTCTTCGCTATT
His-R	GTGGTGGTGGTGGTGGTG

^a^Underlined nucleotides are restriction sites of the enzymes indicated in the brackets at the ends.

### Preparation of tongue sole head kidney lymphocytes (HKLs)

Head kidneys were collected aseptically from three tongue sole (average body weight, 481 ± 52 g). After washing three times with PBS containing 100 U of penicillin and streptomycin (Beijing Solarbio Science & Technology Co., Ltd., Beijing, China), tissues were passed through a metal mesh and the cell suspension was collected. HKLs were then prepared using the Fish Lymphocyte Separation Kit (Hao Yang Biological Manufacture Co., Ltd., Tianjin, China). The prepared HKLs were added to the wells of 96-well tissue culture plates (1 × 10^5^ cells/well) containing L-15 medium (Thermo Scientific HyClone, Beijing, China) and cultured at 22°C.

### Preparation of rat anti-tongue sole IgM antibody

Three tongue sole (average body weight, 690 ± 80 g) were inoculated with *E*. *tarda* TX1 (5 × 10^3^ CFU/fish) via intraperitoneal injection, as described above. The fish were re-inoculated with TX1 at 30 and 45 days after the first inoculation. At 15 days after the last inoculation, blood was collected from the fish and used for serum preparation. Immunoglobulin M (IgM) was prepared from the sera using the IgM Purification Kit (Thermo Fisher Scientific, Inc., Waltham, MA, USA). IgM was mixed with complete Freund’s adjuvant, and the mixture was used to immunize rats. At 30 and 45 days after the first immunization, booster immunizations were performed with IgM mixed with incomplete Freund’s adjuvant. At 60 days after the first immunization, blood was collected from the animals and used for serum preparation. Antibody titer was determined using an enzyme-linked immunosorbent assay (ELISA). The specificity of the antiserum was examined by Western blot analysis as reported previously [28], with tongue sole Epstein-Barr virus-induced gene 3 (CsEBI3) protein as a negative control protein (S1 Fig).

### Microscopic analysis

Immunofluorescence microscopy was performed as described in a previous publication [29]. Briefly, HKLs were prepared as described above and resuspended in PBS to a concentration of 10^7^ cells/ml. Then, rCsBAFF or rGST (10 μg/ml) was added to the cell suspensions. After incubation at 22°C for 1 h, the cells were centrifuged at 300 × *g* for 5 min. The cellular pellets were collected, washed with PBS, resuspended in PBS, and then incubated with mouse anti-His monoclonal antibody (dilution, 1/1000; Tiangen Biotech Co., Ltd.) and rat anti-IgM antibody (prepared above) at 22°C for 1 h. After incubation, the cells were centrifuged as above, resuspended in PBS, and then incubated with fluorescein isothiocyanate (FITC)-labeled goat anti-mouse IgG (dilution, 1/1000; Beijing Biosynthesis Biotechnology Co., Ltd., Beijing, China) and rhodamine B isothiocyanate-labeled goat anti-rat IgG (dilution, 1/1000; Beijing Biosynthesis Biotechnology Co., Ltd.) at 22°C for 1 h. The cells were collected by centrifugation, washed, re-suspended in PBS, and examined with a fluorescence microscope (E800; Nikon Corporation, Tokyo, Japan).

### MTT (3-(4,5-dimethylthiazol-2-yl)-2,5-diphenyltetrazolium) assay

The MTT assay was performed as reported previously [30]. Briefly, HKLs in a 96-well tissue culture plate (1 × 10^5^ cells/well) were treated with or without (control) different concentrations (1, 2, 4, 6, 8, and 10 μg/ml) of rCsBAFF or rGST for 48 h. After treatment, MTT was added to the plate, which was then incubated at 22°C for 4 h. Dimethyl sulfoxide was then added to the plate and the absorption at 490 nm (A_490_) was measured. Results are expressed as proliferation indices, which were calculated as follows: A_490_ of protein-treated cells/A_490_ of untreated control cells. The experiment was performed independently three times.

### Flow cytometry

Flow cytometry was performed as reported previously [23]. Briefly, HKLs were prepared as described above and then placed in the wells of 12-well tissue culture plates (1×10^6^ cells/well) containing L-15 medium supplemented with 10% fetal bovine serum, 100 U/ml of penicillin, and 100 μg/ml of streptomycin. rCsBAFF or rGST was added to the cells to a final concentration of 8 μg/ml, while PBS was added to the control cells. The cells were incubated at 22°C for 36 h, washed with cold PBS, treated with FITC-conjugated annexin V and propidium iodide (PI) using the Annexin V-FITC and PI Cell Apoptosis Detection Kit (Majorbio Biotech Co., Ltd., Shanghai, China), according to the manufacturer’s instructions, and then subjected to flow cytometry using a FACSort Flow Cytometer (BD Biosciences, San Jose, CA, USA) equipped with FlowJo software (Tree Star, Inc., Ashland, OR, USA) for data analysis. The experiment was performed three times.

### CsBAFF overexpression in fish tissues

To construct pCsBAFF, which expresses His-tagged CsBAFF, *CsBAFF* was PCR-amplified with the primer pair BAFF-F2/BAFF-R1 (Table 1), then the PCR products were ligated into the T-A cloning vector pEASY-Simple-T (Beijing TransGen Biotech Co., Ltd., Beijing, China). The recombinant plasmids were digested with the EcoRV restriction endonuclease and the fragment containing *CsBAFF* was recovered and inserted into pCN3 [31] at the EcoRV restriction site. pCsBAFF and pCN3 were diluted in PBS to a concentration of 200 μg/ml. Tongue sole (average body weight, 14.4 ± 0.2 g) received intramuscular injection of 100 μl of pCsBAFF, pCN3, or PBS, as a control. At 7 days post-plasmid injection, tissues (kidney, spleen, and muscle) were collected aseptically. For the detection of plasmids in tissues, tissue DNA was prepared using the TIANamp DNA Kit (Tiangen Biotech Co., Ltd.). PCR was performed with the primer pair CN-F1/CN-R1 (Table 1), which is specific to pCsBAFF and pCN3. For the detection of *CsBAFF* expression carried on the plasmid, tissue RNA was prepared and cDNA was synthesized, as described above, and amplified by RT-PCR with the primer pair BAFF-F3/His-R (Table 1).

### Respiratory burst and acid phosphatase activity

Tongue sole were administered with or without pCsBAFF and pCN3, and at 7 days post-plasmid administration, head kidneys were removed from the fish and washed with PBS. Head kidney macrophage (HKM) preparation and respiratory burst analysis were performed as reported elsewhere [32]. Acid phosphatase activity was determined using the Acid Phosphatase Assay Kit (Beyotime Institute of Biotechnology, Beijing, China). All experiments were performed independently three times.

### CsBAFF overexpression and *E*. *tarda* infection

Tongue sole (average body weight, 14.2 g) were injected with pCsBAFF, pCN3, or PBS (control), as described above. The fish were reared under normal conditions for 7 days and then infected with *E*. *tarda*, as described above. Kidney, spleen, and blood were collected aseptically at 12 and 24 hpi, and bacterial recovery was determined, as reported previously [33]. The experiment was performed independently three times.

### CsBAFF knockdown and *E*. *tarda* infection

*CsBAFF* knockdown was achieved through small interfering RNA (siRNA) technology. To select siRNAs with an interfering effect on CsBAFF expression, three different siRNAs targeting CsBAFF were inserted into pRNAT-CMV3.1 (GenScript, Piscataway, NJ, USA) at the BamHI/AlfII restriction sites, resulting in plasmids psiCsBAFF-1, psiCsBAFF-2, and psiCsBAFF-3. In addition, the plasmid psiCsBAFF-C, which expresses a scrambled siRNA sequence, was similarly constructed. To assess the efficiency of the siRNAs expressed from the plasmids, five groups of tongue sole (n = 5 each) received intramuscular injections of each plasmid (15 μg/fish) or PBS as a control. At 7 days post-plasmid injection, *CsBAFF* expression in kidney and spleen tissues was determined by qRT-PCR, as described above. The plasmid with the strongest inhibitory effect on *CsBAFF* expression was re-named psiCsBAFF. This screening experiment was performed three times. The siRNA sequences expressed by psiCsBAFF and psiCsBAFF-C were 5′-CGCCACATTCATGGGCGCTTTCAAA-3′ and 5′-GTGCCGCACCTTAATACTCGAATCG-3′, respectively. For bacterial infection, tongue sole were administered with psiCsBAFF, psiCsBAFF-C, or PBS (control), as described above. At 7 days post-plasmid injection, the fish were infected with *E*. *tarda*. At 12 and 24 hpi, the numbers of bacteria in blood, kidney, and spleen tissues were determined, as described above. The experiment was performed independently three times.

### Vaccination

pCsBAFF, pCN3, and pCEsa1, a DNA vaccine that expresses the *esa1* gene of *E*. *tarda* [32], were diluted in PBS to concentrations of 100 μg/ml. For preparation of pCEsa1 plus pCsBAFF (pCEsa1 + pCsBAFF), pCEsa1 and pCsBAFF were diluted in PBS to 200 μg/ml and mixed at an equal volume. Tongue sole (average body weight, 14.4 ± 0.2 g) were divided randomly into five groups (n = 45 each) and injected intramuscularly with 100 μl of pCEsa1, pCEsa1 + pCsBAFF, pCsBAFF, pCN3, or PBS (control). At 7 days post-vaccination, expression of the plasmid-coded genes in the vaccinated fish was verified by RT-PCR, as described above. At 30 days post-vaccination, 30 fish from each group were infected via intramuscular injection of *E*. *tarda* (2 × 10^5^ CFU/fish). Mortality was recorded for 20 days, with moribund fish being euthanized, as described above. Bacterial recovery from moribund fish was determined, as described above. The vaccination experiment was conducted twice.

### ELISA

Sera samples were serially diluted two-fold in PBS and serum antibody titers were determined by ELISA, as reported previously [32]. Briefly, 96-well ELISA plates were treated with coating buffer for 1 h and washed with PBST (0.05% Tween-20 in PBS). Then, purified recombinant Esa1 [29], dissolved in coating buffer (10 μg/ml), was added to the plates (100 μl per well). After incubation at 4°C overnight, the plates were washed with PBST, coated with 250 μl of 1% albumin from bovine serum (BSA) per well, and incubated at 22°C for 2 h. Afterward, the plates were washed three times and diluted serum was added to the plates at 100 μl/well. Then, the plates were incubated and washed, as described above. Rat anti-tongue sole IgM antibody (dilution, 1/500) was added to the plates, which were then incubated at 22°C for 1 h. The plates were washed three times, as described above, and horseradish peroxidase-conjugated goat anti-rat IgG (dilution, 1/1000; Beijing Biosynthesis Biotechnology Co., Ltd.) was added to each. The plates were then incubated at 22°C for 1 h. Color development was performed using the Soluble TMB Kit (Tiangen Biotech Co., Ltd.). The plates were read at 450 nm with a precision microplate reader (Molecular Devices, LLC, Sunnyvale, CA, USA). The assay was performed three times.

### Statistical analysis

Other than vaccination, which was performed twice, all experiments were performed three times. Statistical analyses were performed using SPSS 17.0 software (IBM-SPSS. Inc., Chicago, IL, USA). Analysis of variance or, for vaccination, the log-rank test, was used for data analysis. A probability (*p*) value of < 0.05 was considered statistically significant.

## Results

### Characterization of CsBAFF

**Fig 1:**
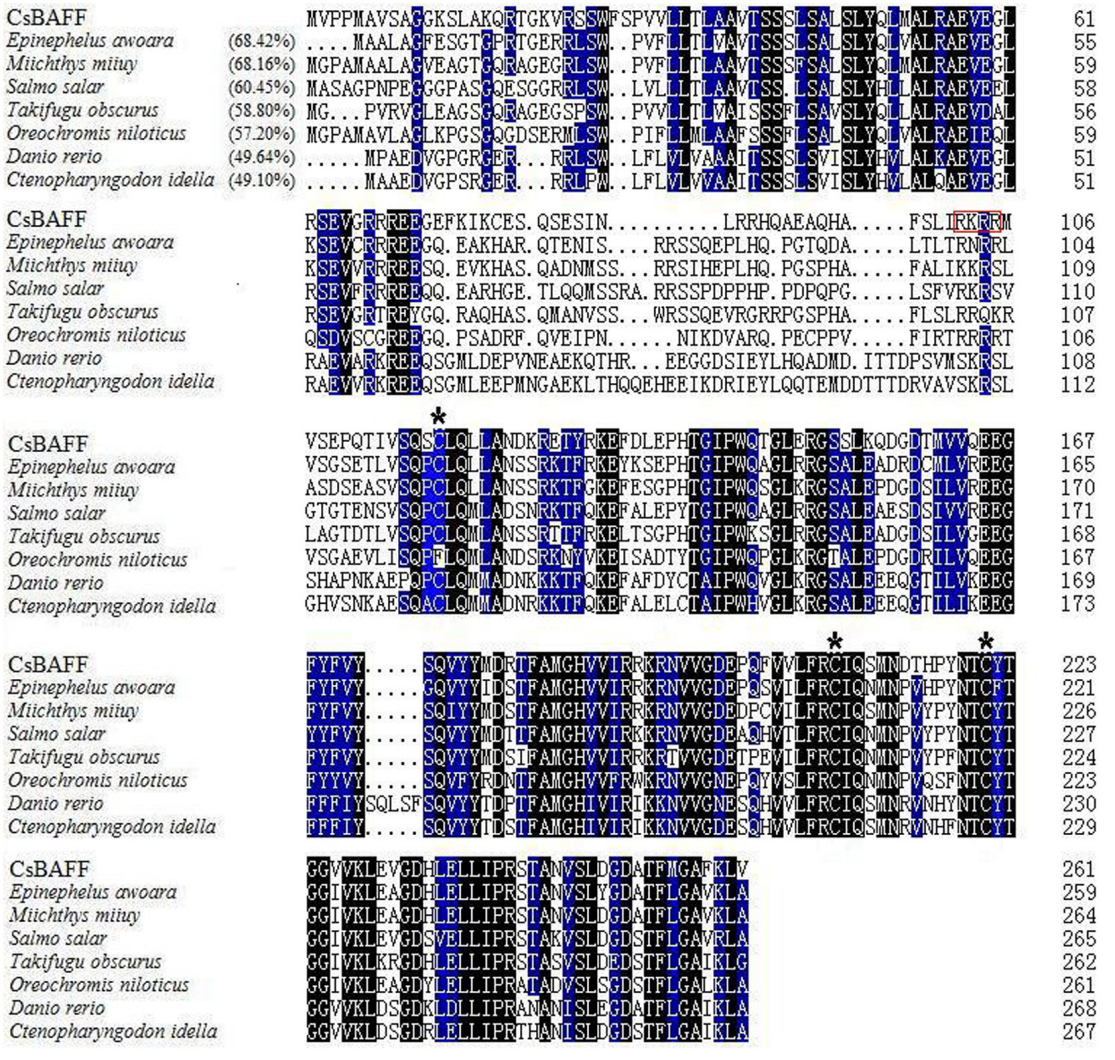
Alignment of the sequences of CsBAFF homologues. Dots denote gaps introduced for maximum matching. Numbers in brackets indicate overall sequence identities between CsBAFF and the compared sequences. The consensus residues are in black, and the residues that are ≥75% identical among the aligned sequences are in blue. The three conserved cysteine residues are indicated by asterisks, and the putative furin cleavage site is boxed in red. The GenBank accession numbers of the aligned sequences are as follows: *Epinephelus awoara*, AFN70720.1; *Miichthys miiu*, AHL44989.1; *Salmo salar*, ACI33633.1; *Takifugu obscurus*, AEB69781.1; *Oreochromis niloticus*, AHF20914.1; *Danio rerio*, NP_001107062.1; *Ctenopharyngodon idella*, AGG11791.1.

CsBAFF consists of 261 amino acid residues and contains a conserved domain typical of the TNF superfamily, which is formed by residues 112–260. A putative furin cleavage site (RKRR), three conserved cysteine residues (Cys^118^, Cys^208^, and Cys^221^), and a conserved long D-E loop known as the “Flap”, which is unique to BAFF in the TNF family [34], were found in CsBAFF (Fig 1). BLAST analysis showed that CsBAFF shares 49.1%–68.4% overall sequence identities with the BAFF homologues of the teleosts *Epinephelus awoara*, *Miichthys miiu*, *Salmo salar*, *Takifugu obscurus*, *Oreochromis niloticus*, *Danio rerio*, and *Ctenopharyngodon idella* (Fig 1).

### CsBAFF expression in the absence and presence of bacterial infection

**Fig 2:**
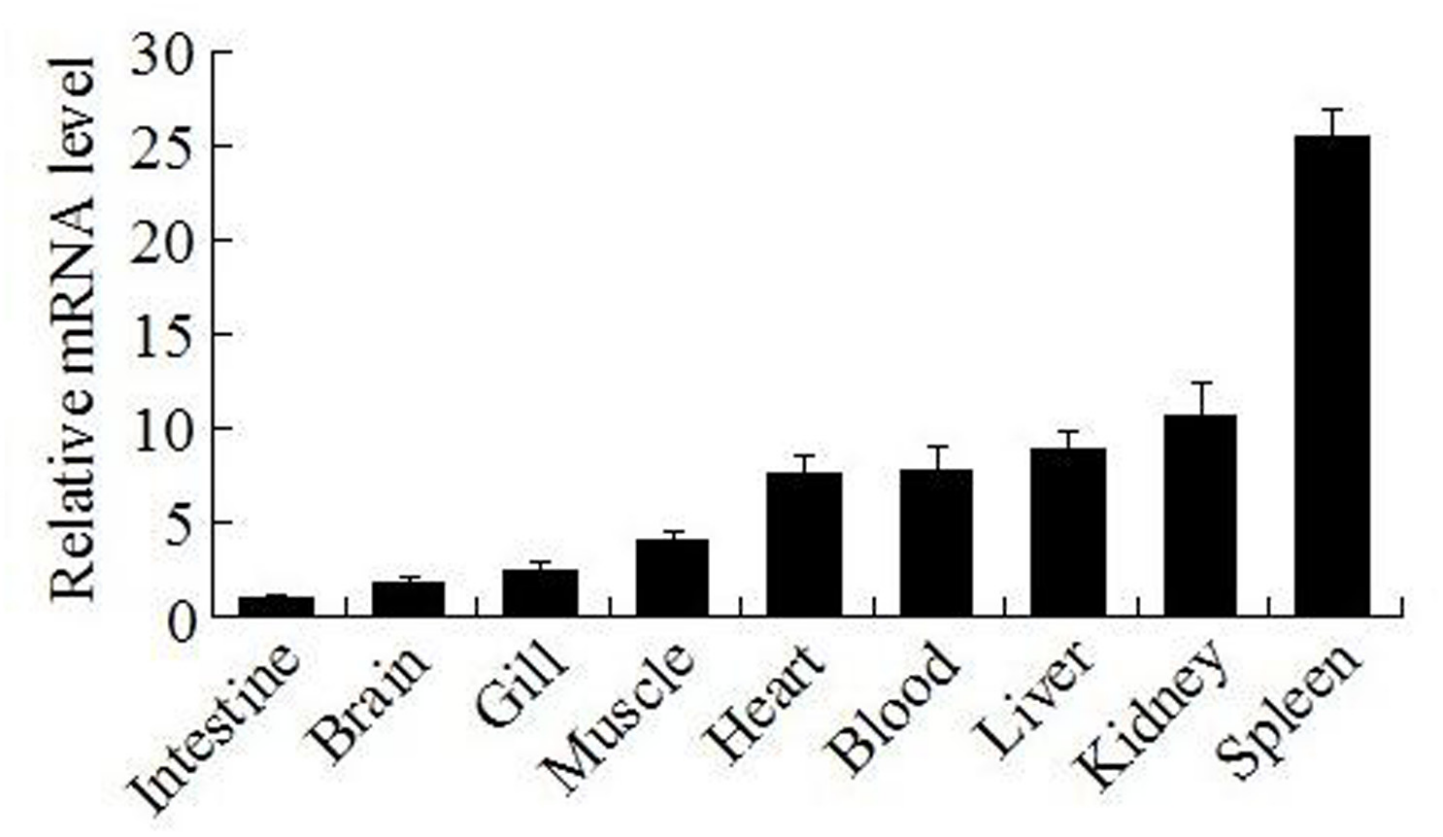
*CsBAFF* expression in fish tissues under normal physiological condition. *CsBAFF* expression in the intestine, brain, gill, muscle, heart, blood, liver, kidney, and spleen was determined by quantitative real time RT-PCR. For the convenience of comparison, the expression level of *CsBAFF* in intestine (Ct value of 8.9) was set as 1. Vertical bars represent means ± SEM (N = 3). N, the number of times the experiment was performed.

**Fig 3:**
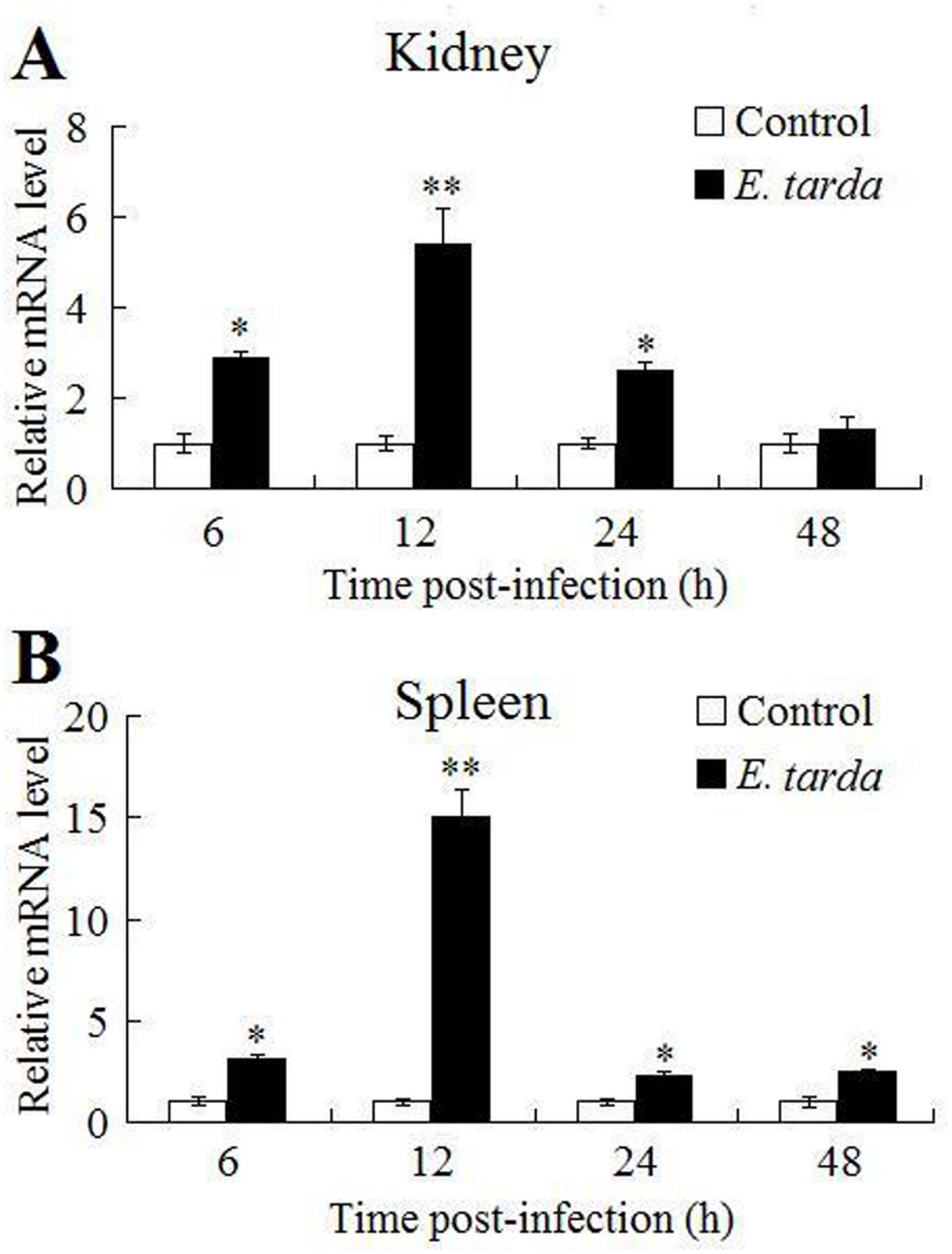
*CsBAFF* expression in response to bacterial infection. Tongue sole were infected with or without (control) *Edwardsiella tarda*, and *CsBAFF* expression in kidney (A) and spleen (B) was determined by quantitative real time RT-PCR at various time points. In each case, the expression level of the control fish was set as 1. The mean Ct values of control fish were 6.4, 6.1, 6.3 and 5.7 at 6 h, 12 h, 24 h, and 48 h respectively in kidney, and 4.3, 4.6, 4.2 and 3.9 at 6 h, 12 h, 24 h, and 48 h respectively in spleen. Values are shown as means ± SEM (N = 3). N, the number of times the experiment was performed. **P < 0.01, *P < 0.05.

qRT-PCR analysis showed that in the absence of bacterial infection, *CsBAFF* expression levels, in increasing order, were greatest in the intestine, brain, gill, muscle, heart, blood, liver, kidney, and were lowest in the spleen (Fig 2). Following infection with the bacterial pathogen *E*. *tarda*, *CsBAFF* expression in the kidney was significantly upregulated at 6, 12, and 24 hpi, with peak expression (5.4-fold) occurring at 12 hpi (Fig 3A). Similar expression profiles were observed in the spleen (Fig 3B).

### Binding of rCsBAFF to lymphocytes and its effect on cellular proliferation and survival

**Fig 4:**
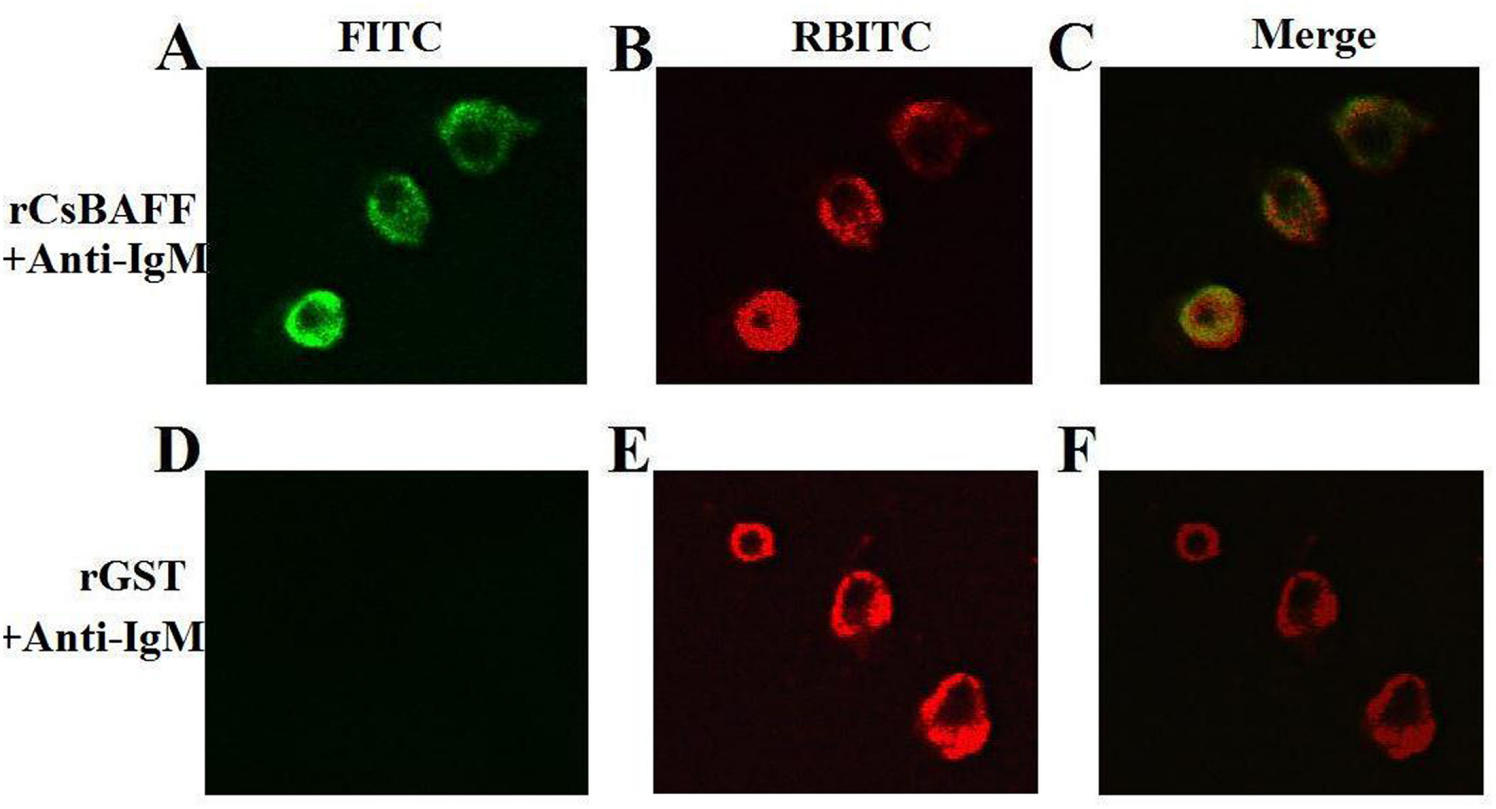
Binding of rCsBAFF to lymphocytes. Tongue sole lymphocytes were incubated with rCsBAFF (A and B) or rGST (D and E), and the cells were treated with FITC-labeled antibody (A and D, for detecting His-tagged protein) or RBITC-labeled antibody (B and E, for detecting cell-surface IgM). The cells were observed under a fluorescence microscope. C, merge of A and B; F, merge of D and E. Magnification, 400 ×.

**Fig 5:**
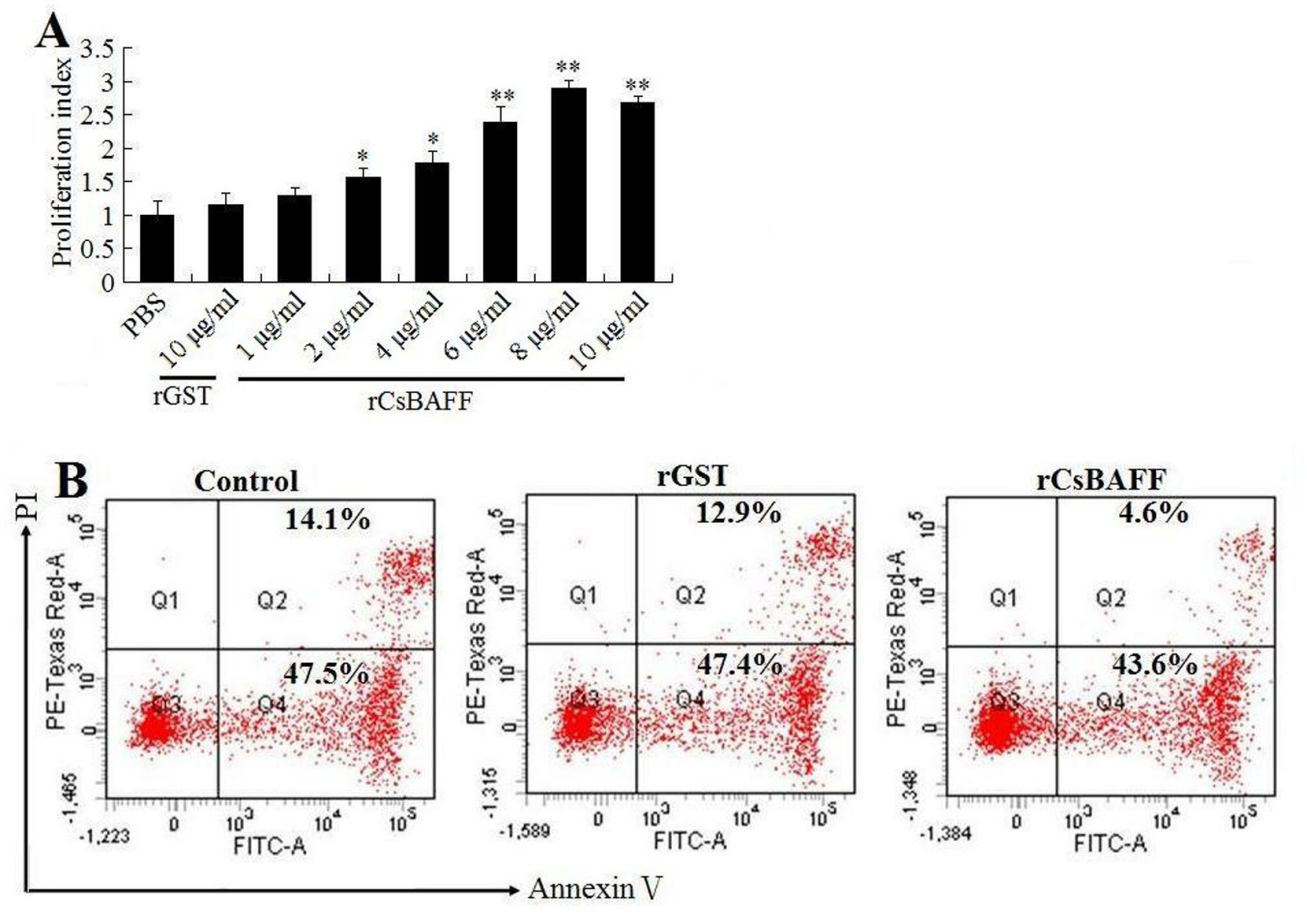
Effect of rCsBAFF on the proliferation and survival of lymphocytes. (A) Tongue sole lymphocytes were incubated with different concentrations of rCsBAFF or rGST, and cellular proliferation was determined by MTT assay. Data are presented as means ± SEM (N = 3). *N*, the number of times the experiment was performed. *P < 0.05; **P < 0.01. (B) Tongue sole lymphocytes were incubated with or without (control) 8 μg/ml rCsBAFF or rGST and stained with Annexin V and PI; the cells were then analyzed by flow cytometry. The results are one representative of three experiments.

In order to investigate the biological activity of CsBAFF, His-tagged rCsBAFF was purified from *E*. *coli* (S2 Fig). As a control, rGST was purified under the same conditions. Immunofluorescence microscopy revealed that following the incubation of rCsBAFF or rGST with lymphocytes, rCsBAFF, but not rGST, was detected on the cells (Fig 4), suggesting binding of the protein to the lymphocytes. Furthermore, rCsBAFF-bound cells also reacted with anti-IgM antibody, suggesting that these cells were likely B cells. The MTT assay results showed that the interaction of rCsBAFF with lymphocytes enhanced cell proliferation in a dose-dependent manner (Fig 5A). To determine whether rCsBAFF had any effect on lymphocyte survival, cells were treated with rCsBAFF or rGST, and cellular apoptosis was evaluated by flow cytometry after annexin V/PI staining. The results showed that rCsBAFF treatment significantly reduced the number of apoptotic lymphocytes, as compared to that of control cells (48.2% vs. 61.6%, respectively; Fig 5B). In contrast, the frequency of apoptosis (60.3%) of lymphocytes treated with rGST was comparable to that of the control cells.

### Effect of CsBAFF overexpression on the immune response of tongue sole

#### Overexpression of CsBAFF

To overexpress CsBAFF, tongue sole were administered with pCsBAFF, which expresses His-tagged CsBAFF, or the control plasmid pCN3. At 7 days post-administration, PCR detected pCsBAFF and pCN3 in the muscle, kidney, and spleen tissues of fish administered with the respective plasmids, but not in those administered with PBS (S3A Fig and data not shown). In the same tissues, RT-PCR detected mRNA specific to pCsBAFF-encoded *CsBAFF* in pCsBAFF-administered fish, but not in pCN3- or PBS-administered fish (S3B Fig and data not shown). These results indicate that the *CsBAFF* gene coded by pCsBAFF was successfully expressed *in vivo*.

#### Effect of CsBAFF overexpression on macrophage activation

**Fig 6:**
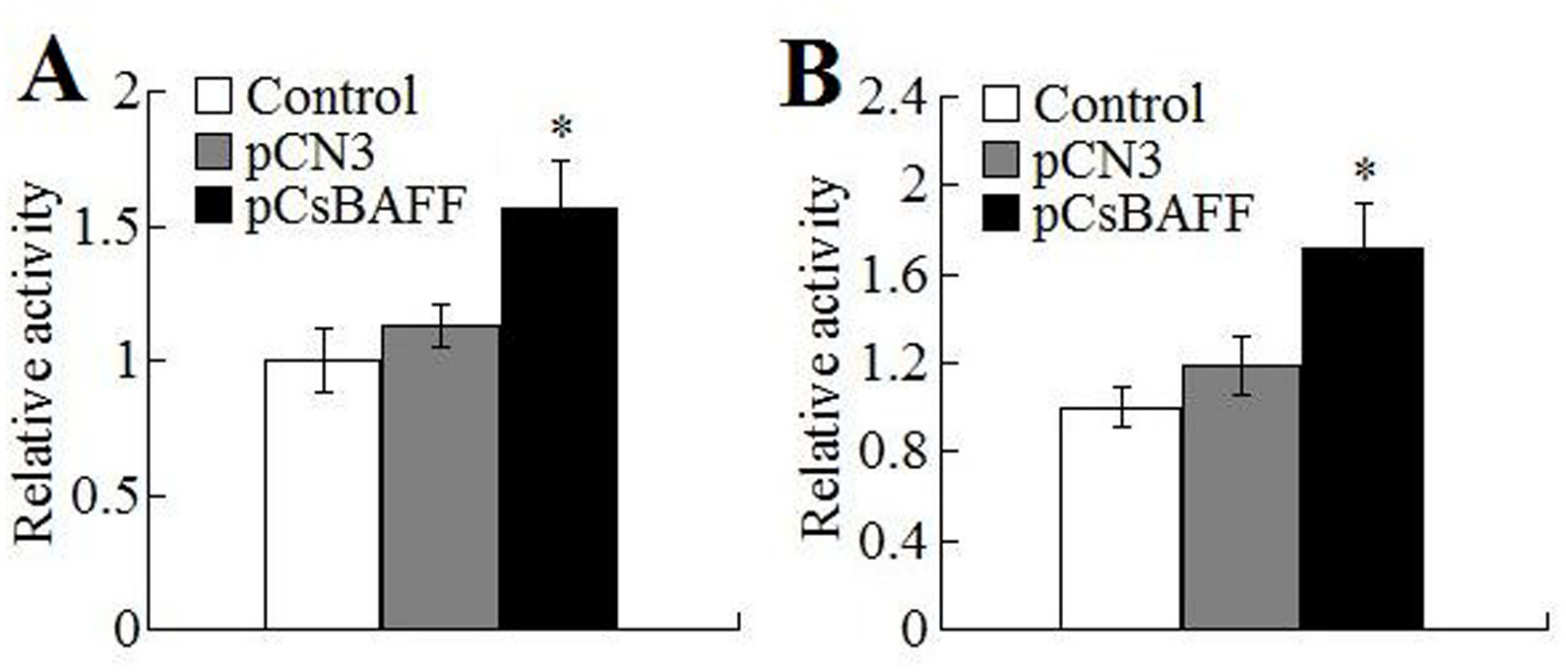
Effect of CsBAFF overexpression on macrophage activation. Macrophages from tongue sole administered with pCsBAFF, pCN3, and PBS (control) were examined for respiratory burst activity (A) and acid phosphatase activity (B). The respiratory burst activity and phosphatase activity are shown as values relative to those of the control cells (*A*_630_ of 0.46 and *A*_410_ of 0.7 respectively). Data are expressed as means ± SEM (N = 3). N, the number of times the experiment was performed. *P < 0.05.

Immune response analysis showed that, compared to macrophages from the fish administered with PBS, macrophages from those administered with pCsBAFF exhibited significantly enhanced respiratory burst activity and acid phosphatase activity (Fig 6). In contrast, macrophage activities from pCN3-administered fish were comparable to those of the control fish.

#### Effect of CsBAFF overexpression on bacterial infection

**Fig 7:**
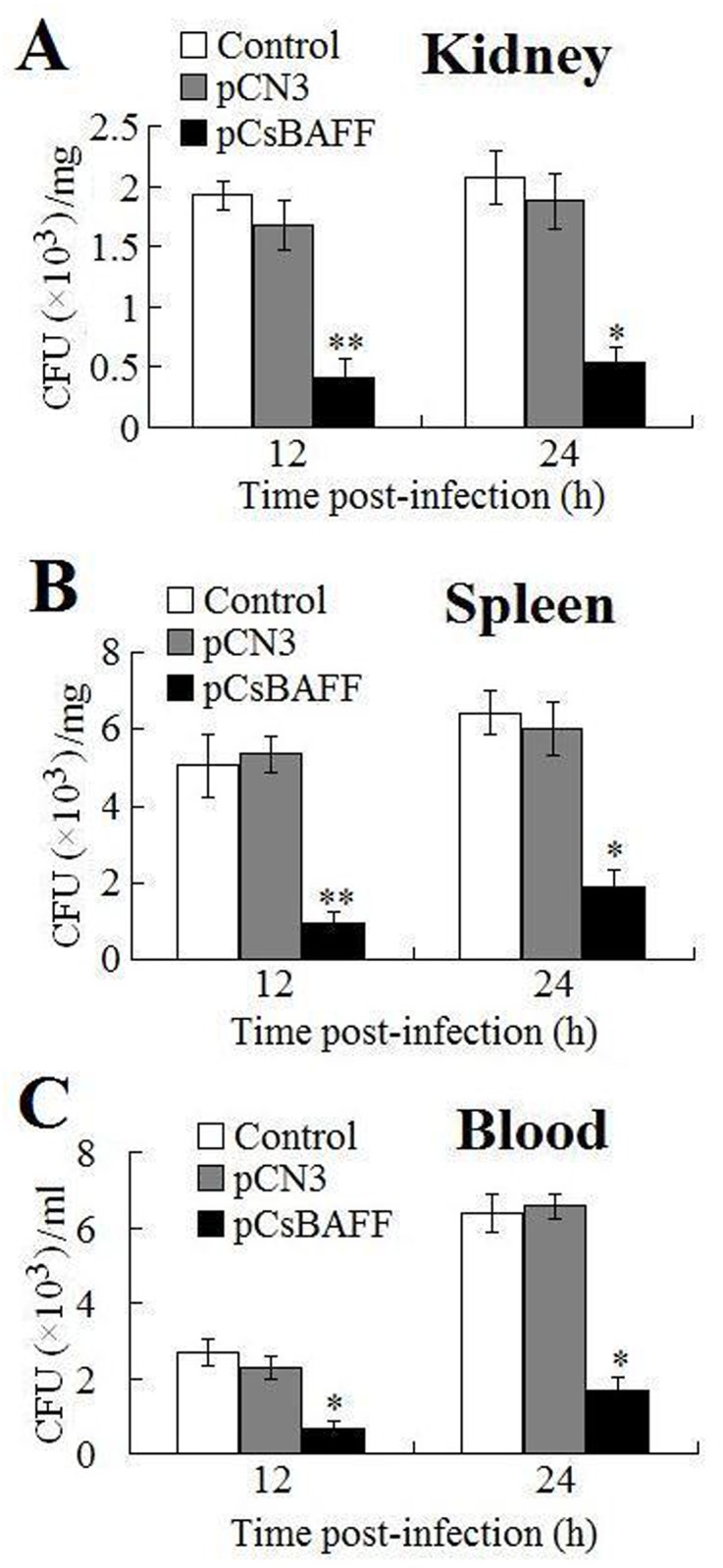
Effect of CsBAFF overexpression on bacterial infection. Tongue sole administered with pCsBAFF, pCN3, or PBS (control) were infected with *Edwardsiella tarda*, and the amount of bacteria in kidney (A), spleen (B), and blood (C) was determined at different time points. Data are expressed as means ± SEM (N = 3). N, the number of times the experiment was performed. **P < 0.01, *P < 0.05.

To examine the effect of CsBAFF overexpression on bacterial infection, fish were infected with *E*. *tarda* at 7 days post-plasmid administration and the bacterial load in blood, kidney, and spleen tissues was determined at 12 and 24 hpi. The results showed a significantly lower number of bacteria in all examined tissues of pCsBAFF-administered fish than in the control fish, whereas the numbers of bacteria in pCN3-administered fish were comparable to those in the control fish (Fig 7).

### *CsBAFF* knockdown and its effect on bacterial infection

#### *CsBAFF* knockdown

We further investigated the involvement of CsBAFF in antibacterial immunity by knocking down *CsBAFF* expression via RNA interference. Briefly, tongue sole were administered with psiCsBAFF, which expresses a *CsBAFF*-targeting siRNA, and *CsBAFF* expression in kidney and spleen tissues was determined by qRT-PCR at 7 days post-plasmid administration. The results showed that *CsBAFF* expression in both tissues of fish administered with psiCsBAFF was significantly reduced, as compared to that of the control fish (S4 Fig). In contrast, *CsBAFF* expression in tongue sole administered with the plasmid psiCsBAFF-C, which expresses a nonspecific siRNA, was comparable to that in the control fish.

#### Effect of *CsBAFF* knockdown on bacterial infection

**Fig 8:**
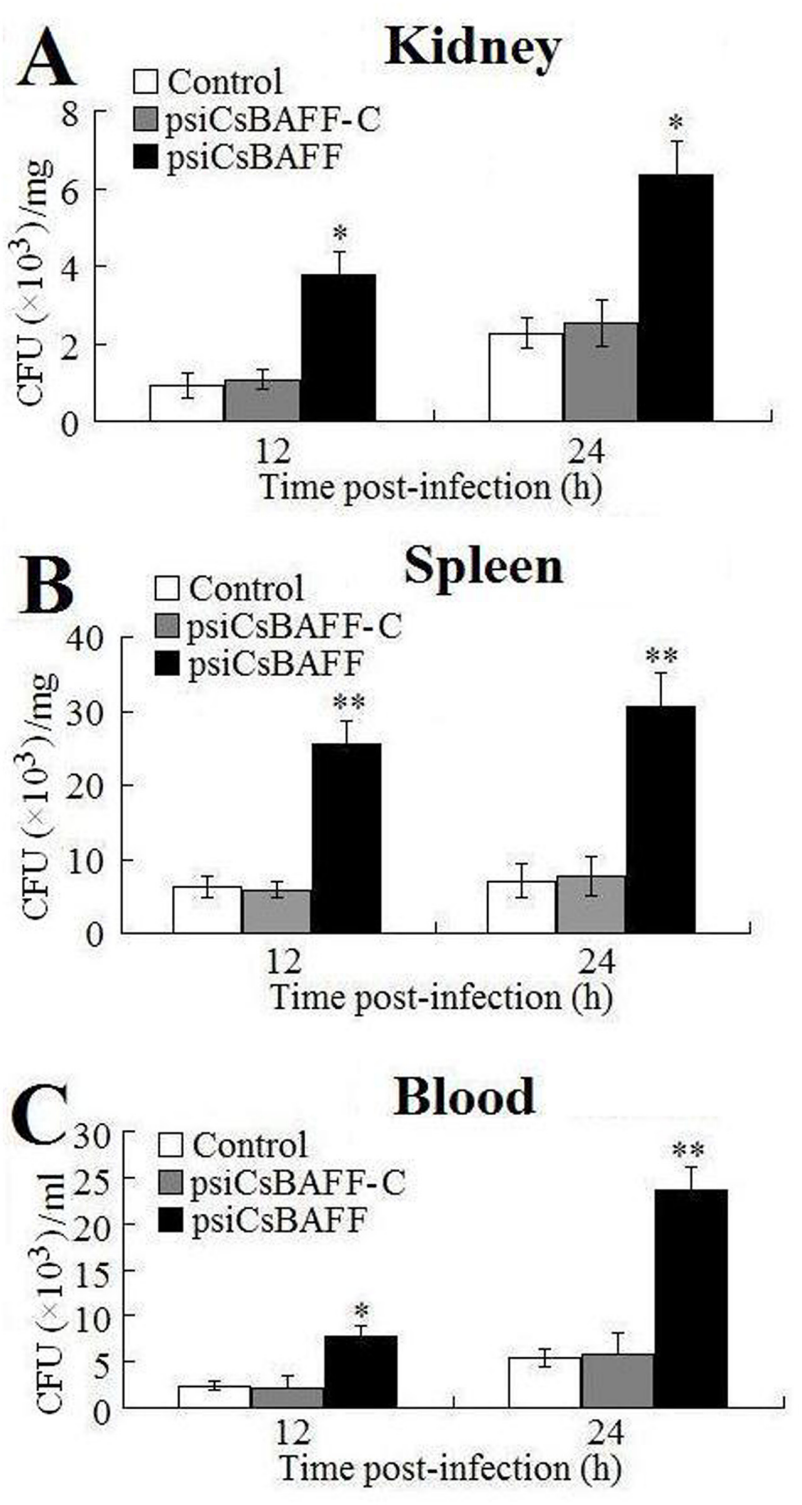
Effect of CsBAFF knockdown on bacterial infection. Tongue sole administered with psiCsBAFF, psiCsBAFF-C, or PBS (control) were infected with *Edwardsiella tarda*, and the amount of bacteria in kidney (A), spleen (B), and blood (C) was determined at different time points. Data are expressed as the mean ± SEM (N = 3). N, the number of times the experiment was performed. **P < 0.01; *P < 0.05.

Tongue sole administered with psiCsBAFF and psiCsBAFF-C were infected with *E*. *tarda* and the bacterial numbers in blood, kidney, and spleen tissues were determined at 12 and 24 hpi. The results showed that in all examined tissues and at both time points, the bacterial numbers in fish administered with psiCsBAFF were significantly higher than those in the control fish, whereas the bacterial numbers in fish administered with psiCsBAFF-C were comparable to those in the control fish (Fig 8).

### Effect of CsBAFF on the protective immunity of a DNA vaccine

#### Vaccination of tongue sole

Given the immune effect observed above with CsBAFF, we wondered whether CsBAFF conveyed any promoting effect on the protective immunity of vaccines. To investigate this question, tongue sole were vaccinated with pCEsa1 (an *E*. *tarda*-targeting DNA vaccine that expresses the *esa1* gene of *E*. *tarda*) or pCEsa1 plus pCsBAFF (pCEsa1 + pCsBAFF). For comparison, fish were also vaccinated with pCsBAFF and pCN3 alone. At 7 days post-vaccination, expression of plasmid-encoded genes in fish tissues was verified by RT-PCR, which showed that *esa1* and *CsBAFF* were detected in the muscle, kidney, and spleen tissues of fish vaccinated with pCEsa1 and pCsBAFF, respectively, while both *esa1* and *CsBAFF* were detected in the fish vaccinated with pCEsa1 + pCsBAFF (S5 Fig and data not shown). In contrast, *esa1* or *CsBAFF* expression was not detected in pCN3-vaccinated fish.

#### Protective effect of the vaccines

**Fig 9:**
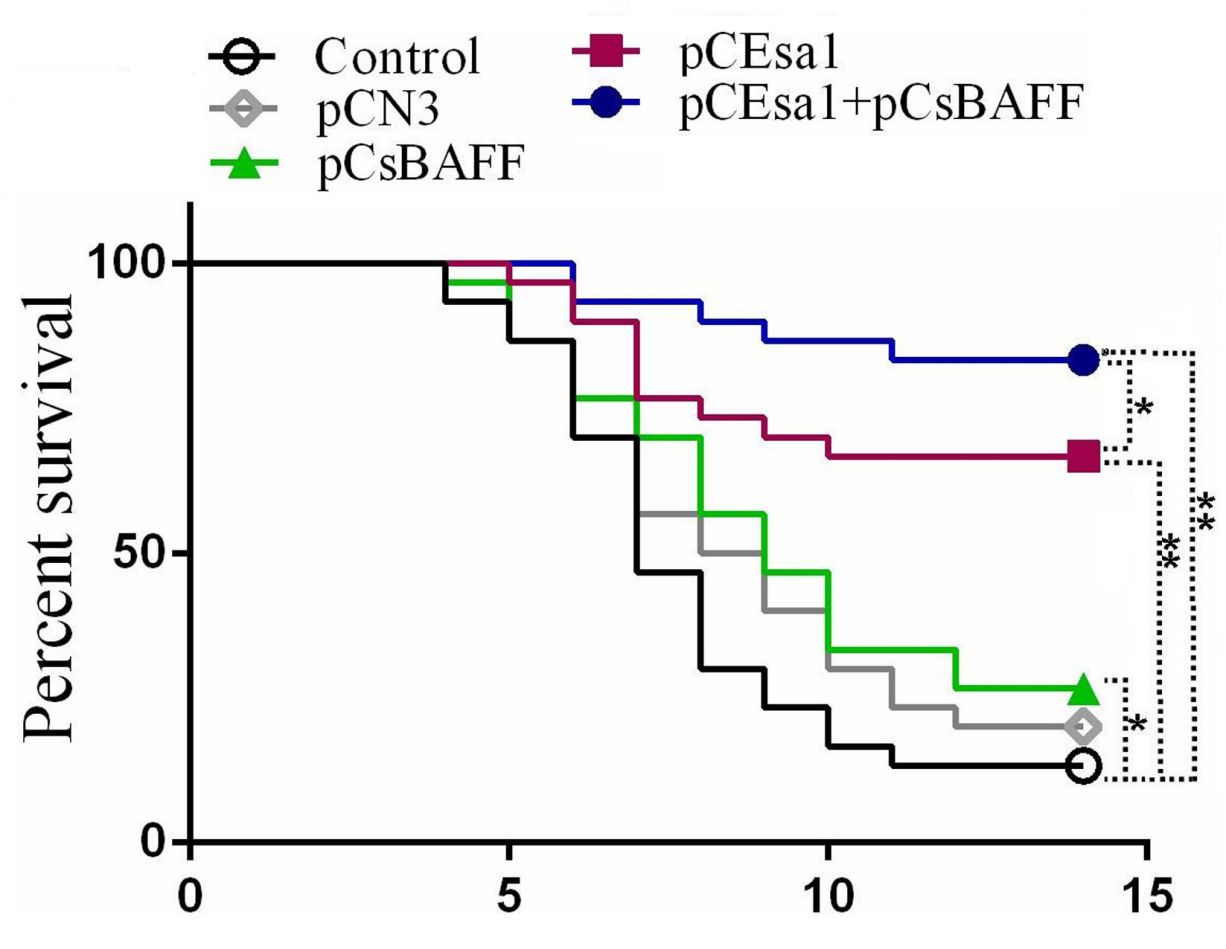
Survival of vaccinated fish. Tongue sole vaccinated with or without (control) pCEsa1, pCEsa1 plus pCsBAFF, pCsBAFF, or pCN3 were challenged with *Edwardsiella tarda* and monitored daily for survival. The results are one representative vaccination trial. Significance between the survivals of the vaccinated fish and control fish was determined with logrank test. **P < 0.01, *P < 0.05.

Fish were challenged with *E*. *tarda* at one month after vaccination. The survival rates of fish vaccinated with pCEsa1, pCEsa1 + pCsBAFF, pCsBAFF, and pCN3 were 66.7%, 83.3%, 26.7%, and 20%, respectively, while the survival rate of the control fish was 13.3% (Fig 9). Statistical analysis showed that the survival rate of the fish vaccinated with pCEsa1 + pCsBAFF was significantly greater than that of fish vaccinated with pCEsa1. Comparable results were obtained in a second vaccination trial. Bacterial recovery analysis showed that *E*. *tarda* was detected in the kidney, spleen, and liver tissues of dying fish. ELISA analysis showed that the level of serum antibodies against Esa1 in the fish vaccinated with pCEsa1 + pCsBAFF was significantly higher than that in pCEsa1-vaccinated fish (S6 Fig).

## Discussion

In this study, we analyzed the expression levels and biological properties of a teleost BAFF, CsBAFF, in tongue sole. CsBAFF possesses the domain structure of the TNF superfamily and contains the three cysteine residues and the “Flap” structure found in all BAFFs. These features indicate that CsBAFF is a member of the BAFF family. In higher vertebrates, BAFF is known to exist in the form of either a transmembrane protein or a soluble protein, the latter being the result of cleavage by a furin-like protease. In teleost BAFF, this cleavage site is preserved in some species, but not in others, such as zebrafish, tetrodon, and rainbow trout. In the case of CsBAFF, the putative furin cleavage site was identified, suggesting that CsBAFF is likely a soluble protein.

Tissue-specific BAFF expression has been reported in both mammals and fish. In mammals, relatively high *BAFF* transcription levels were found in the spleen [35–38]. Abundant *BAFF* expression was also reported in the spleen tissues of Japanese sea perch, fugu, yellow grouper, grass carp, and rainbow trout [20–22,39,40]. Similarly, the results of the present study showed that *CsBAFF* expression was highest in the spleen and abundant in kidney, and that *E*. *tarda* infection caused a significant induction of *CsBAFF* expression in both kidney and spleen tissues. In teleosts, the spleen and kidneys are the primary sources of B and T lymphocytes [41]; therefore, these results support a role for CsBAFF in the immune response of tongue sole.

Immunofluorescence microscopy showed that rCsBAFF was able to bind HKLs, suggesting that CsBAFF is probably a soluble molecule that, as observed in mammalian counterparts, functions by interaction with its receptors on target cells, which is in agreement with the presence of the furin cleavage site. In higher vertebrates, BAFF is known to be a survival factor for immature and mature B cells and induces activation of the nuclear factor κB (NF-κB) pathway, as well as upregulates expression of pro-survival molecules, such as myeloid cell leukemia 1 and B-cell lymphoma-extra-large [15,42,43]. The results of the present study showed that in the presence of rCsBAFF, HKLs exhibited significantly elevated proliferative activity and reduced the apoptosis rate. These results indicate that, as with mammalian BAFF, rCsBAFF is able to promote lymphocyte proliferation and survival.

Studies in mammals have shown that BAFF plays a role in the differentiation of DCs and monocytes into activated macrophages, which secrete a variety of cytokines and chemokines, such as interleukin (IL)-6, IL-10, IL-12, cyclooxygenase-2, and type I interferons [44–48]. Macrophages are more easily activated in a cytokine-rich environment [49]. In the present study, *in vivo* analysis showed that in fish administered with the CsBAFF-expressing plasmid pCsBAFF, significant inductions of respiratory burst and acid phosphatase activities were observed in HKMs, suggesting that CsBAFF overexpression induced macrophage activation. In line with these results, following *E*. *tarda* infection, the bacterial loads in pCsBAFF-administered fish were significantly lower than those in the control fish, most likely due to the enhanced activation of macrophages, which promoted clearance of the invading pathogens.

Inhibition of BAFF expression is known to have a strong impact on the immune response of mammals. Studies with BAFF-deficient mice and BAFF-transgenic mice demonstrated that BAFF is essential for both B cell homeostasis and the regulation of B cell selection, while the lack of BAFF results in inhibition of B-cell development and antibody response [50–56]. Furthermore, BAFF repression impairs the antibody response to T cell-dependent antigens and decreased capacity to clear bacterial infection [57,58]. In our study, we found that tongue sole administered with psiCsBAFF exhibited significantly reduced *CsBAFF* expression and, following *E*. *tarda* infection, enhanced bacterial invasion into the kidney, spleen, and blood. These results, which are in contrast to the observations of CsBAFF overexpression, further support to the role of CsBAFF in the antibacterial immune response of tongue sole.

BAFF, which is involved in activation of DCs and T cells, has been investigated as a vaccine adjuvant in several mammalian studies [59–62], which reported that BAFF modulated the differentiation of memory B cells to plasma cells and that BAFF exerted a potent co-stimulatory effect and enhanced CD4+ and CD8+ T cell responses [12–14]. In our study, the potential effect of CsBAFF on the DNA vaccine pCEsa1 was examined. We found that as compared to vaccination with pCEsa1, vaccination with pCEsa1 + pCsBAFF increased the survival rate by ~17% and, consistently, fish vaccinated with pCEsa1 + pCsBAFF exhibited a significantly higher level of specific serum antibodies than those vaccinated with pCEsa1 alone. These results indicate that pCsBAFF enhanced the specific immune response induced by pCEsa1.

In conclusion, the results of our *in vivo* and *in vitro* studies demonstrated that CsBAFF stimulates macrophage activation and promotes lymphocyte proliferation/survival, and that normal CsBAFF expression in tongue sole is required for optimal antibacterial immunity. Furthermore, CsBAFF, when co-administered with a DNA vaccine, augments the specific immunity induced by the vaccine. These observations indicate for the first time that teleost BAFF plays a significant immunostimulatory role *in vivo*, which adds further insights into the biological function of teleost BAFF.

## Supporting Information

S1 Fig Western blot analysis of the specificity of IgM antiserum. Purified tongue sole IgM (lane 3) and recombinant CsEBI3 (lane 1) were subjected to Western blot using IgM antiserum. Lane 2, protein markers.
(TIF)

S2 Fig SDS-PAGE analysis of rCsBAFF and rGST. Purified rCsBAFF (lane 2 of A) and rGST (lane 2 of B) were analyzed by SDS-PAGE and viewed after staining with Coomassie brilliant blue R-250. Lane 1, protein markers.
(TIF)

S3 Fig Detection of plasmids (A) and expression of plasmid-derived *CsBAFF* (B) in tongue sole. (A) Tongue sole were administered with pCN3, pCsBAFF, and PBS (lanes 2, 3, and 4 respectively) for 7 days. DNA was extracted from spleen and used for PCR with primers specific to the common region in pCsBAFF and pCN3. (B) Tongue sole were administered with pCsBAFF (lane 2), pCN3 (lane 3), and PBS (lane 4) for 7 days. RNA was extracted from spleen and used for RT-PCR with primers specific to plasmid-derived *CsBAFF* (upper panel), or, as an internal control, to β-actin (lower panel). Lane 1 of both panels, DNA molecular weight markers.
(TIF)

S4 Fig Expression of *CsBAFF* in psiCsBAFF-administered fish. Tongue sole were administered with psiCsBAFF, psiCsBAFF-C, or PBS (control), and *CsBAFF* expression in kidney and spleen was determined by quantitative real time RT-PCR at 7 days post-plasmid administration. In both tissues, the expression level of the control fish was set as 1. Values are shown as means ± SEM (N = 3). N, the number of times the experiment was performed. *P < 0.05.
(TIF)

S5 Fig Expression of the vaccine gene in vaccinated fish. Tongue sole were vaccinated with pCEsa1 + pCsBAFF (lanes 2 and 4), pCsBAFF (lane 3), pCEsa1 (lane 5), and pCN3 (lanes 6 and 7). At 7 days post-vaccination, RNA was extracted from spleen and used for RT-PCR with primers specific to plasmid-derived *CsBAFF* (lanes 2, 3, and 6), *esa1* (lanes 4, 5, and 7), or, as an internal control, to β-actin (B). Lane 1 of both panels, DNA molecular weight markers.
(TIF)

S6 Fig Serum antibody production in vaccinated fish. Sera were taken from tongue sole vaccinated with pCEsa1, pCEsa1 plus pCsBAFF, and PBS (control) for one month. Serum antibodies against Esa1 were determined by enzyme-linked immunosorbent assay. Values are shown as mean ± SEM (N = 3). N, the number of times the assay was performed. **P < 0.01; *P < 0.05.
(TIF)
