# Supplementary figures and images for: CsBAFF, a Teleost B Cell Activating Factor, Promotes Pathogen-Induced Innate Immunity and Vaccine-Induced Adaptive Immunity
Source: PLoS One. 2015 Aug 21;10(8):e0136015. doi: 10.1371/journal.pone.0136015 (PMC4546598; doi:10.1371/journal.pone.0136015)

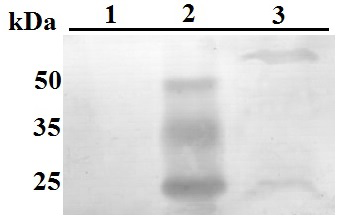

Supplement: S1 Fig — Purified tongue sole IgM (lane 3) and recombinant CsEBI3 (lane 1) were subjected to Western blot using IgM antiserum. Lane 2, protein markers. (TIF) [file pone.0136015.s001.tif]

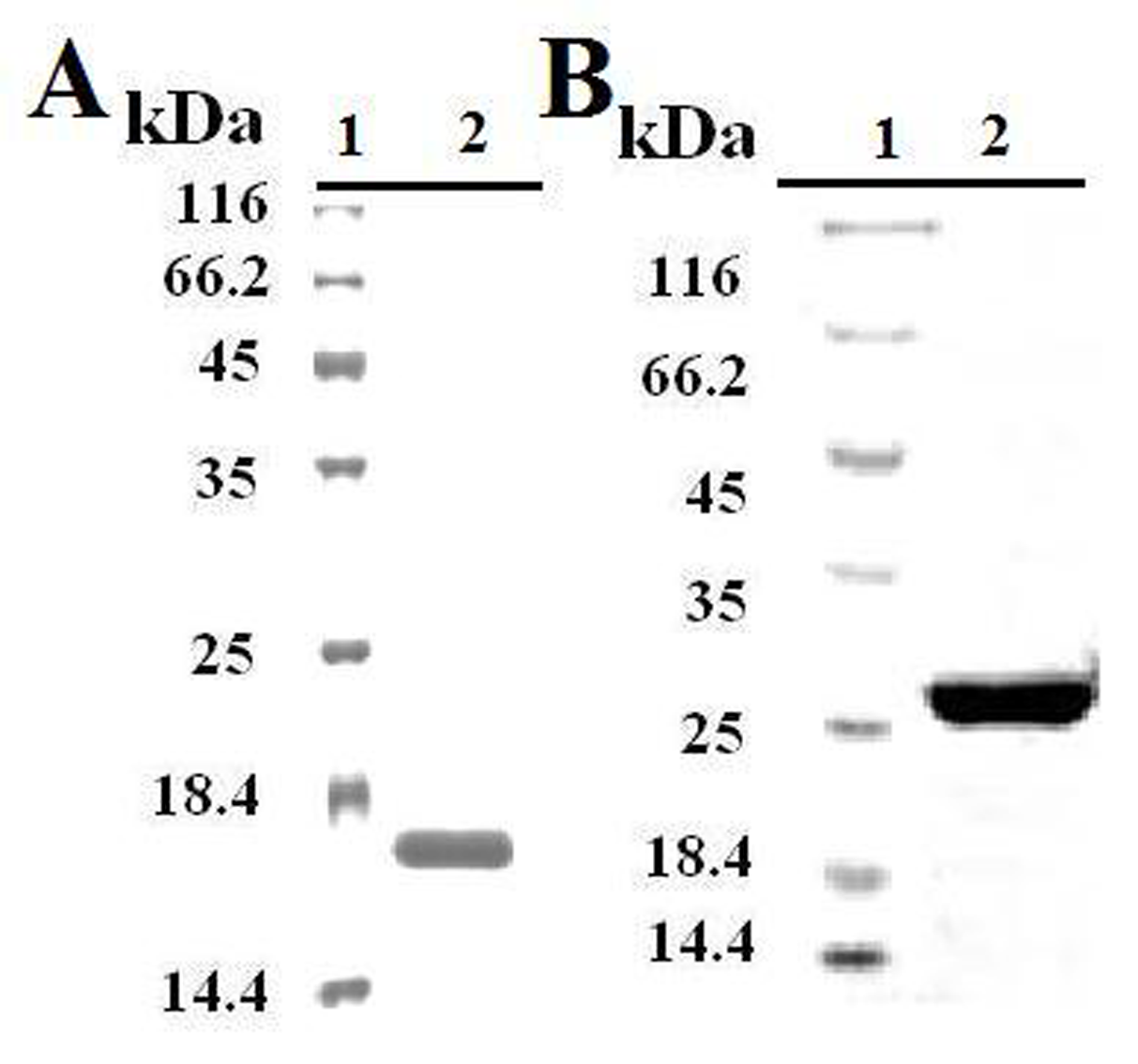

Supplement: S2 Fig — Purified rCsBAFF (lane 2 of A) and rGST (lane 2 of B) were analyzed by SDS-PAGE and viewed after staining with Coomassie brilliant blue R-250. Lane 1, protein markers. (TIF) [file pone.0136015.s002.tif]

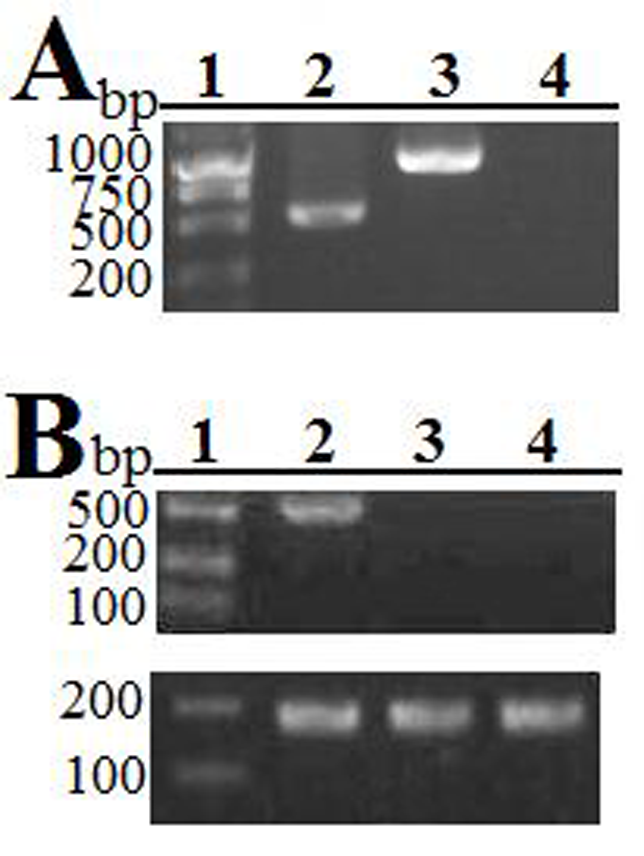

Supplement: S3 Fig — (A) Tongue sole were administered with pCN3, pCsBAFF, and PBS (lanes 2, 3, and 4 respectively) for 7 days. DNA was extracted from spleen and used for PCR with primers specific to the common region in pCsBAFF and pCN3. (B) Tongue sole were administered with pCsBAFF (lane 2), pCN3 (lane 3), and PBS (lane 4) for 7 days. RNA was extracted from spleen and used for RT-PCR with primers specific to plasmid-derived CsBAFF (upper panel), or, as an internal control, to β-actin (lower panel). Lane 1 of both panels, DNA molecular weight markers. (TIF) [file pone.0136015.s003.tif]

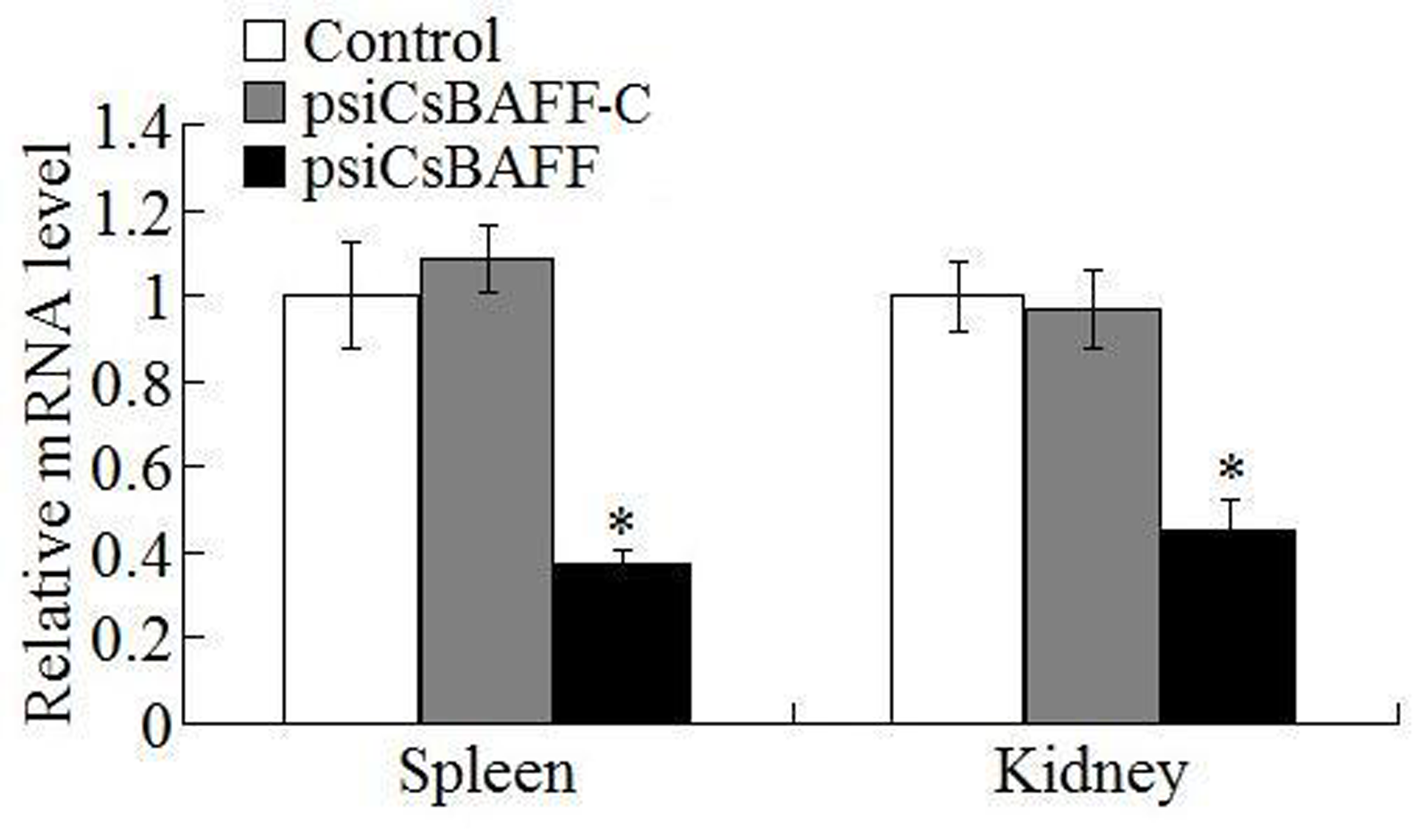

Supplement: S4 Fig — Tongue sole were administered with psiCsBAFF, psiCsBAFF-C, or PBS (control), and CsBAFF expression in kidney and spleen was determined by quantitative real time RT-PCR at 7 days post-plasmid administration. In both tissues, the expression level of the control fish was set as 1. Values are shown as means ± SEM (N = 3). N, the number of times the experiment was performed. *P < 0.05. (TIF) [file pone.0136015.s004.tif]

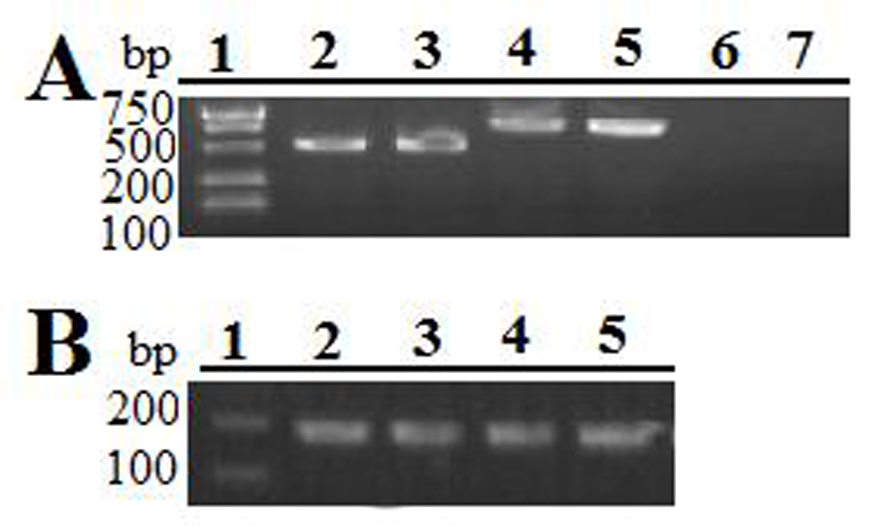

Supplement: S5 Fig — Tongue sole were vaccinated with pCEsa1 + pCsBAFF (lanes 2 and 4), pCsBAFF (lane 3), pCEsa1 (lane 5), and pCN3 (lanes 6 and 7). At 7 days post-vaccination, RNA was extracted from spleen and used for RT-PCR with primers specific to plasmid-derived CsBAFF (lanes 2, 3, and 6), esa1 (lanes 4, 5, and 7), or, as an internal control, to β-actin (B). Lane 1 of both panels, DNA molecular weight markers. (TIF) [file pone.0136015.s005.tif]

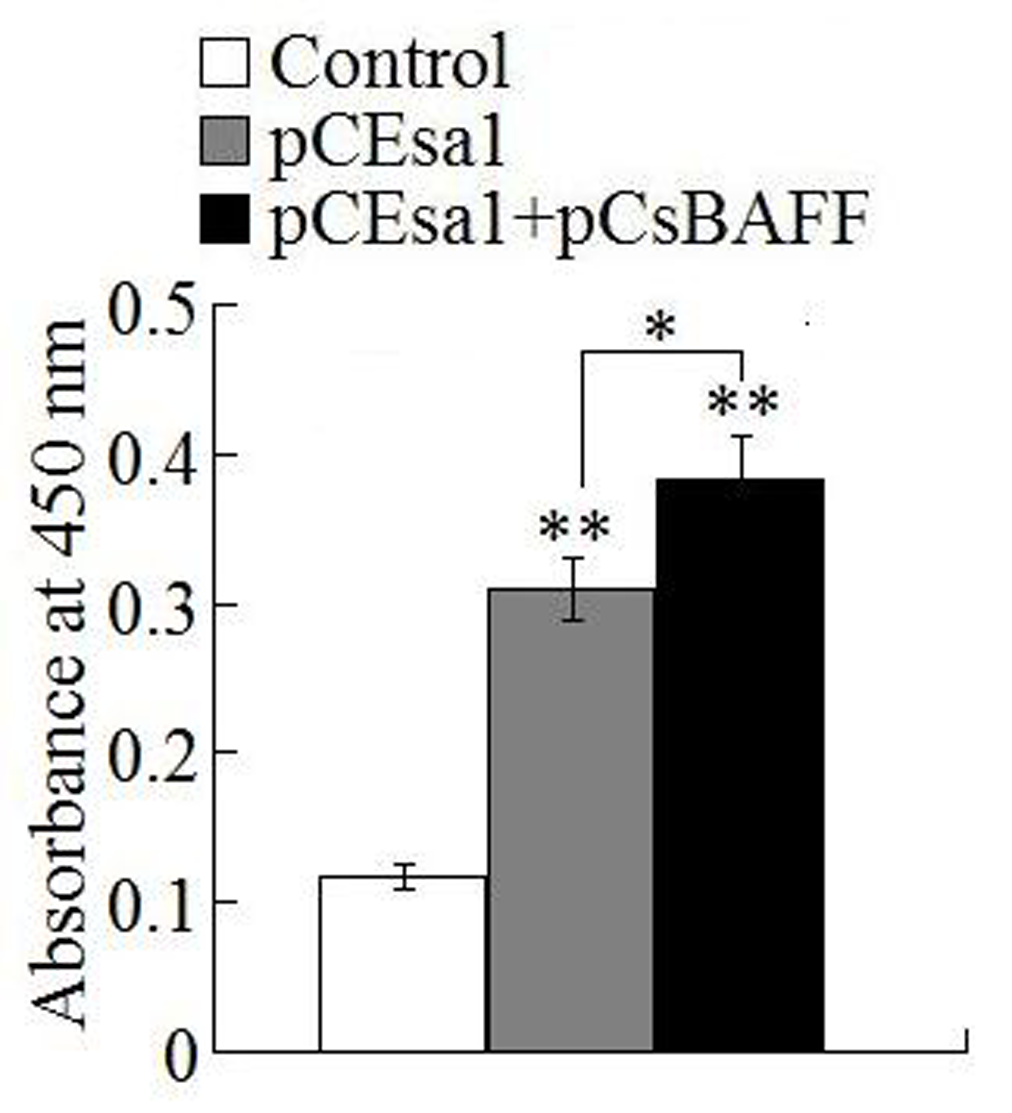

Supplement: S6 Fig — Sera were taken from tongue sole vaccinated with pCEsa1, pCEsa1 plus pCsBAFF, and PBS (control) for one month. Serum antibodies against Esa1 were determined by enzyme-linked immunosorbent assay. Values are shown as mean ± SEM (N = 3). N, the number of times the assay was performed. **P < 0.01; *P < 0.05. (TIF) [file pone.0136015.s006.tif]
